# Looking Beyond Th17 Cells: A Role for Tr1 Cells in Ankylosing Spondylitis?

**DOI:** 10.3389/fimmu.2020.608900

**Published:** 2020-12-02

**Authors:** Joanna McGinty, Nicola Brittain, Tony J. Kenna

**Affiliations:** School of Biomedical Sciences, Queensland University of Technology, Brisbane, QLD, Australia

**Keywords:** ankylosing spondylitis, inflammation, T cells, regulatory T cells, Tr1 cells, genomics

## Introduction

The chronic inflammatory arthritis ankylosing spondylitis (AS) is a highly heritable disease of complex genetics (1–3). The genetic association between AS and *HLAB27* has been studied for almost 50 years, and over the last decade or so large-scale genomics studies have defined variants outside the *HLA* that confer risk of developing AS (1, 2, 4). Key among these are genes involved in T cell activation (including *ERAP1, ERAP2, NPEPPS, UBE2E3, UBE2L3*), immune signaling (including *IL23R, IL6R, IL12B, TYK2*) and various transcription factors involved in functional differentiation of immune cells (including *TBX21, RUNX3 and EOMES*). Functional genomic approaches have implicated several immune cell types in disease processes, and studies support a role for microbial dysbiosis in disease pathogenesis. It is now obvious that AS is not only a genetically complex but also immunologically complex disease. To date, however, much effort has focused on ‘low hanging fruit’ from genomics studies, most notably *IL23R*. This important work has led to advances in treatment options for AS patients through inhibiting the pathogenic effects of IL-17. But trials of the IL-23 inhibitor Ustekinumab were not successful. Potential reasons for the failure of Ustekinumab Phase 3 trials in AS are many and include difficulties with outcome measures and trial design. But the failure has also sparked the community to re-evaluate subtleties in models of AS immunopathogenesis.

With many AS-associated genes implicated in various aspects of T cell biology, it is hard to pinpoint exact processes or pathways that are of critical importance in AS. Speculation needs to be supported by empirical observations from well-designed studies that push us beyond consideration of Th17 cell biology. Recently, Hanson and colleagues (5) provided evidence that AS patients exhibit significant reductions in the size of CD4 and CD8 T cell expansions globally in the peripheral blood, suggesting that perturbations in T cell survival, senescence, or regulation of clonal proliferation occur in AS patients during adaptive immune responses. This brings into focus what role regulatory T cells play in AS and indeed which populations of regulatory T cells may be of relevance to AS.

Regulatory T cells were originally described as a subset of immune cells critical for negative regulation of immune-mediated inflammation and prevention of autoimmune diseases. However, Tregs are also implicated in the suppression of both innate and adaptive immune cells towards allergens, organ transplants, commensals, food, and other innocuous environmental triggers (6).

FoxP3^+^ Tregs may be thymically induced (tTregs), peripherally induced (pTregs) or induced in cell culture, in response to TGF-*β*. The tissue environment promotes Tregs to express tissue-specific transcription factors that cooperate with FoxP3, providing a specialized function and supporting Treg cell subset homeostasis (7). Tregs regulate their immune environment by contact-dependent mechanisms, such as CD95 induction of conventional T cell apoptosis and CTLA4 downregulation of APC co-stimulatory function, as well as cytokine-mediated functions, including CD25 adsorption of IL-2 and IL-10 secretion which attenuates DC function and promotes Tr1 cell differentiation (8).

Tr1 cells, another subtype of regulatory T cells, do not constitutively express the transcription factor Foxp3. Upon TCR recognition of their cognate antigen at the site of tissue inflammation, Tr1 cells secrete large quantities of IL-10, which has many immunomodulatory effects on local immune cells (9). Both Tr1 and Treg cells serve a vital role in preventing deleterious immune responses with comparable mechanisms of suppression, yet Tregs are essential in the initial stage of immune suppression at the site of inflammation, while Tr1 cells are key for the maintenance of long-term tolerance and restoration of tissue homeostasis (10).

## Tr1 Cells are Important Regulators of Inflammation

Tr1 cells were first described in severe combined immunodeficiency disease (SCID) in the early 1990’s (11). Since then, accumulating evidence implicates impaired function and reduced Tr1 cell numbers in immunopathogenesis of various immune-mediated diseases. Among these are inflammatory bowel disease (IBD), psoriasis, multiple sclerosis (MS), Grave’s disease, Hashimoto’s thyroiditis, and systemic lupus erythematosus (12–14). Broadly, IL-10 produced by Tr1 cells is a key regulator of TNF-mediated pathologies (15).

**Figure 1:**
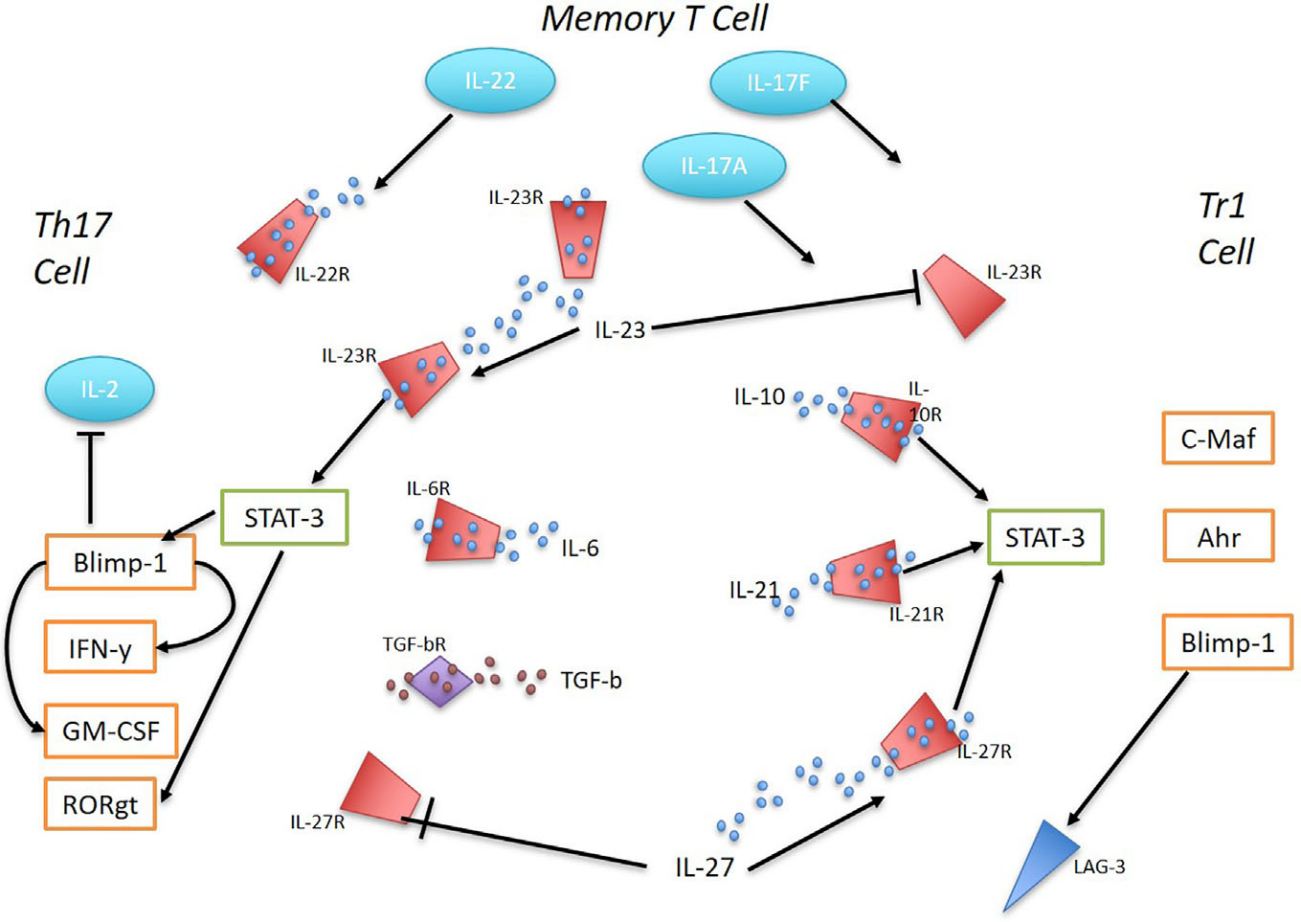
The interplay between Th17 and Tr1 cells in inflammation. Ahr, c-Maf, and Blimp-1 promote the differentiation of Tr1 cells in response to IL-10, IL-21, and IL-27 *via* STAT3 activation. IL-27 was found to be sufficient to induce Blimp-1 in Tr1 cells and sustain high IL-10 production. IL-23 supresses Tr1 cells. Early Th17 cell development is initiated by TGF-B and IL-6, inducing IL-23R expression and IL-17 production. IL-23 signaling regulates Th17 cells and their pathogenicity. STAT-3 mediated IL-23-dependent Blimp-1 enhances Th17 pathogenic factors by increasing IL-23R expression, GM-CSF, IFN-y and repressing IL-2. ROR*γ*t expression is stabilized by IL-23R expression. IL-27 supresses Th17 cells. Memory T cells in response to TLR signaling produce IL-23. Memory T cells with Th17 pro-inflammatory phenotype secrete large amounts of 17A, IL-17F and IL-22.

Naïve CD4+ T cells acquire a Tr1 phenotype upon cytokine signaling *via* IL-10, IL-21, or IL-27 which promotes STAT3 activation and subsequent priming of the *IL10* locus. Transcription factors that bind to *IL10* include EOMES, IRF4, c-Maf, Ahr, and Blimp-1 which act through multiple pathways to induce a stable production of IL-10 (10). In contrast, Th17 cells, although necessary for host defense against extracellular pathogens, when dysregulated become major pathogenic drivers of inflammation in many immune-mediated diseases. TGF-*β* and IL-6 are the key cytokines for initiating Th17 differentiation, which induces IL-23R expression as well as high secretion of the pro-inflammatory cytokine IL-17 (16, 17) (**Figure 1**). Microarray gene expression analysis comparing Tr1 cells and Th17 cells prior to IL-23 signaling identified the most predominantly overexpressed genes in Tr1 cells to be *IRF1, IRF8*, *PRDM1* (Blimp-1), and *TBX21* (18). IL-23 is secreted by various immune cells including dendritic cells (DCs) and macrophages in response to toll-like receptor signaling (19). Under homeostatic conditions, the presence of IL-23 in the distal small bowel promotes a localized cytokine environment that targets IL-23 sensitive intestinal cells which support mucosal barrier function and intestinal immunity. LAG-3, which is expressed on natural regulatory T cells (Tregs), induced Tregs and Tr1 cells, has been shown to control intestinal IL-23 production by immunosuppression of CX3CR1+ tissue-resident macrophages and innate lymphoid cells (ILCs) type 3 (19, 20). *In vivo*, IL-23R signaling suppresses the differentiation of FoxP3+ Tregs and Tr1 cells and stabilizes the expression of ROR*γ*t, the Th17 signature transcription factor (16). IL-23 is a key factor for perpetuating and stabilizing Th17 cell activation and cytokine production as it induces strong proliferation of memory T cells that secrete large amounts of IL-17A, IL-17F, and IL-22 (16). IL-23-dependent signaling in Th17 cells induces Blimp-1 and in concert with T-bet, promotes pathogenicity by upregulating IL-23R expression GM-CSF and IFN-y while repressing IL-2 in a STAT3-mediated manner (21, 22).

Recent literature marks dysfunctional Tr1 cells and their reduced capacity to secrete large amounts of immune-mediating cytokine IL-10, as attributing to persistent inflammation in autoimmune disease contexts. Tr1 dysfunction is associated with inflammation in diseases genetically and clinically relevant to AS. For example, Tr1-derived IL-10 has a non-redundant role in preventing gut inflammation in IBD (22). In a mouse model of IBD, *IL23r* mRNA expression is detectable on both Tr1 cells and Th17 cells. Tr1 cells are responsive to IL-23 and downregulate IL-10 in response to IL-23R signaling (22). Clinical and genetic overlap between AS and IBD has been recognized for many years, and an increase in IL-23 has been well documented in both diseases (23, 24). Psoriasis, frequently concomitant with AS, is driven by chronic activation of autoreactive Th17 cells (25). Psoriasis patients exhibit an inverse relationship between disease severity and Tr1 and Treg cell numbers. Tr1 cells were not found in the skin of healthy controls; however, Tr1 cells were identified in the non-lesioned skin of psoriasis patients. Psoriatic lesions revealed an increase in activated CD3^+^CD4^+^CD69^+^ T cells and a lack of Tr1 cells (26).

## The Potential Role of the IL-12/IL-23 and IL-27 Axis in AS Immunopathology

IL-27 signaling induces Blimp-1-mediated IL-10 production in Tr1 cells, and in the absence of IL-23 signaling, Th17 cells respond to IL-27 and IL-12 signaling by secreting IL-10 in a Blimp-1 dependent manner. This demonstrates a potential for plasticity in Th17 cells as they lose their pathogenicity and adopt a Tr1-like phenotype which can contribute to homeostasis under certain conditions (18). In contrast, Th17 cells further stimulated by IL-23 demonstrated a commitment to the inflammatory phenotype (27), known to be implicated in many autoinflammatory conditions including AS (23). IL-27 levels are reported to be elevated in AS patients and to correlate with disease activity measures (28) which would seem to support development of Tr1 cells in AS patients. However, IL-23 counteracts IL-27 and IL-12-mediated effects on Tr1-development reinforcing the pro-inflammatory phenotype of Th17 cells (18). The balance between IL-23 *vs* IL-12/IL-27 signaling in CD4+ effector T cells determines whether tissue inflammation is perpetuated or resolved. It is our opinion that the immunomodulatory function of regulatory T cells is impaired in the context of AS, resulting in perpetual inflammation in the enthesis and ileum of patients with active disease. It is hypothesized that deficient IL-27 signaling, reduced IL-10 production by Tr1 cells and exacerbated IL-23 signaling promotes persistent IL-17 and other pro-inflammatory cytokine production and proliferation of pro-inflammatory T cell subsets. These three key factors that may diminish the capacity for immune regulation are linked to known AS genetic risk factors including *IL27, IL23R, TBX21*, and *EOMES* susceptibility mutations (1, 2). However, the link between AS susceptibility loci and development of Tr1 cells in AS has been further complicated recently. Pepelyayeva and colleagues reported reduced numbers of Tr1 cells in Erap−/− mice (29), a genotype that GWAS data associates with AS protection rather than risk (1). A better understanding of the role, if any, Tr1 cells play in AS patients may be a valuable step towards understanding how to control inflammation in this disease.

## Can Tr1 Cells Offer Any Therapeutic Potential in AS?

It is clear that Tr1 cells are an important regulator of general immune responses. Therapeutic manipulation of Tr1 cells, *ex vivo* or *in vivo* might be highly advantageous in several T cell-mediated diseases. Much progress has already been made in animal models, which proved that Tr1 cell-based therapies may be a feasible approach to treating inflammatory disorders in general.

Adoptive transfer of *in vitro* induced Ag-specific Tr1 cells efficiently prevents colitis induced in SCID mice by pathogenic T cells (30). In a pre-clinical model of type 1 diabetes Tr1 cells induced in the intestinal mucosa migrate to the periphery and control effector T cell responses and the development of diabetes (31). Studies in MS attributed deficiencies in IL-10-secreting Tr1 cells to decreased IL-27 and disruption of the CD46 pathway that promotes transformation of IFN-y-secreting Th1 cells into Tr1 cells. Exogenous IL-27 partially restored the number and function in a mouse model of MS, experimental autoimmune encephalomyelitis (EAE) (32). IL-27 induced Tr1 cells have been described to have therapeutic potential in several autoimmune contexts by expanding their immunomodulatory function in active disease states.

Robust protocols have been established to generate clinical-grade human Tr1 cells (33, 34) and Tr1 cell-based therapies have been trialed in graft *versus* host disease (35) and refractory Crohn’s disease (36) clinical trials.

## Discussion

Tr1 cells have an important role in autoimmune disease prevention, and investigating their potential to restore immune homeostasis in environments of persistent inflammation may be beneficial in the context of AS. While the genetics of AS implicate Tr1 biology in disease processes discrepancies exist between *in vitro* data that suggest the cytokine environment in AS may be suitable for expansion of Tr1 cells (28) and *in vivo* data that show reduced Tr1 cells in mice that lack an important AS-susceptibility gene (29). A challenge from here is to define where and how Tr1 cells may be important in AS and to define pre-clinical models that will allow pre-clinical evaluation of their therapeutic potential in AS.

## Author Contributions

All authors contributed to the article and approved the submitted version.

## Funding

TK is supported by an NHMRC Project grant (APP1162767).

## Conflict of Interest

The authors declare that the research was conducted in the absence of any commercial or financial relationships that could be construed as a potential conflict of interest.
